# Glutamine, but not Branched-Chain Amino Acids, Restores Intestinal Barrier Function during Activity-Based Anorexia

**DOI:** 10.3390/nu11061348

**Published:** 2019-06-15

**Authors:** Clément L’Huillier, Marine Jarbeau, Najate Achamrah, Liliana Belmonte, Asma Amamou, Séverine Nobis, Alexis Goichon, Emmeline Salameh, Wafa Bahlouli, Jean-Luc do Rego, Pierre Déchelotte, Moïse Coëffier

**Affiliations:** 1 UNIROUEN, INSERM UMR 1073 “Nutrition, Inflammation and Gut-Brain Axis”, Normandie University, 76183 Rouen, France; cle.lhuillier@gmail.com (C.L.); marine.jarbeau@univ-rouen.fr (M.J.); najate.achamrah@chu-rouen.fr (N.A.); liliana.belmonte@chu-rouen.fr (L.B.); asma.amamou@etu.univ-rouen.fr (A.A.); severine.nobis@univ-rouen.fr (S.N.); alexis.goichon@univ-rouen.fr (A.G.); emmeline.salameh@etu.univ-rouen.fr (E.S.); wafa.bahlouli@etu.univ-rouen.fr (W.B.); pierre.dechelotte@chu-rouen.fr (P.D.); 2 Institute of Research and Innovation in Biomedicine (IRIB), UNIROUEN, Normandie University, 76183 Rouen, France; jean-luc.do-rego@univ-rouen.fr; 3 Department of Nutrition, Rouen University Hospital, 76183 Rouen, France; 4 Animal Behavior Facility, SCAC, UNIROUEN, 76183 Rouen, France

**Keywords:** glutamine, Branched-chain amino acids, Activity-Based Anorexia, Intestinal permeability

## Abstract

Background: During activity-based anorexia (ABA) in mice, enhanced paracellular permeability and reduced protein synthesis have been shown in the colon while the gut–brain axis has received increasing attention in the regulation of intestinal and mood disorders that frequently occur during anorexia nervosa, a severe eating disorder for which there is no specific treatment. In the present study, we assessed the effects of oral glutamine (Gln) or branched-chain amino acids (BCAA) supplementation during ABA to target intestinal functions, body composition and feeding behavior. Methods: C57BL/6 male mice were randomized in Control (CTRL) and ABA groups. After ABA induction, mice received, or not, either 1% Gln or 2.5% BCAA (Leu, Ile, Val) for one week in drinking water. Results: Neither Gln nor BCAA supplementation affected body weight and body composition, while only Gln supplementation slightly increased food intake. ABA mice exhibited increased paracellular permeability and reduced protein synthesis in the colonic mucosa. Oral Gln restored colonic paracellular permeability and protein synthesis and increased the mucin-2 mRNA level, whereas BCAA did not affect colonic parameters. Conclusion: In conclusion, oral Gln specifically improves colonic response during ABA. These data should be further confirmed in AN patients.

## 1. Introduction

Anorexia nervosa (AN) belongs to eating disorders (ED), as described in the fifth edition of *Diagnostic and Statistical Manual of Mental Disorders* (DSM-V) [1]. AN is a multifactorial disease in which young women are predominantly represented, as shown in a recent study [2]. Considering DSM-V, AN is mainly characterized by severe weight loss (BMI <18.5 kg·m^−2^), persistence of the inability to change behaviors in a way that resolves the weight loss and the lack of recognition of the seriousness of body weight. Mortality in AN is around 1–4% [3] due to its comorbidity with other mental disorders such as anxiety and depression [4]. In addition, physical hyperactivity frequently occurs in anorectic patients [5] and strongly contributes to the energy imbalance affecting all organs such as the heart [6], kidneys [7] and/or bones [8]. Functional digestive disorders also frequently occur in AN patients with delayed gastric emptying, altered motility and increased transit time, abdominal pain, and modification of gut microbiota [9,10], while the role of the microbiota–gut–brain axis in the regulation of feeding and mood disorders has been underlined. Gut microbiota dysbiosis has been reported in AN patients [11] but intestinal permeability remains poorly documented. Monteleone et al. showed reduced small intestinal permeability [12] and Mörkl et al. recently reported that 35.29% of AN patients had increased serum zonulin, a marker of intestinal permeability [13]. In mice, colonic permeability was increased but not jejunal permeability [14].

Refeeding of AN patients that is usually performed through the enteral route via a nasogastric tube [15] only partially restores those parameters. Indeed, studies evaluating body composition changes after refeeding underlined increased truncal fat mass [16] and some studies have shown that intestinal disorders are still present after body weight gain [17], as well as mood disorders [18]. Consequently, innovative nutritional interventions could be of interest to improve refeeding by targeting specific metabolic pathways [19].

In the activity-based anorexia model (ABA) in mice, we previously reported in the colon an altered barrier function [14], a TLR-4 activation [20] and a reduced protein synthesis associated with specific modifications of proteome [21]. Glutamine is a conditionally essential amino acid and the main energy substrate for rapidly dividing cells [22,23]. Many studies report an improvement of intestinal barrier function [24], a reduced intestinal inflammatory response [25,26] and a stimulation of protein synthesis [27] after glutamine supplementation. Glutamine also improves the immune system and prevents bacterial translocation from intestinal lumen to inner layers when administrated in patients under parenteral nutrition [28]. Interestingly, intestinal permeability and abdominal pain were improved by oral glutamine supplementation in a randomized double-blind clinical trial in patients with irritable bowel syndrome (IBS) [29]. Intestinal paracellular permeability is mainly regulated by tight junctions involving claudin proteins (CLDN), occludin (OCDN) or zonula occludens-1 (ZO-1).

Branched chain amino acids (BCAA; Leucine, Isoleucine and Valine) have been proposed to maintain muscle mass or function in different conditions such as intensive physical activity [30,31] or in elderly patients [32]. BCAA are preferentially metabolized in skeletal muscle rather than in liver [33] and are involved in muscle mass development in mice [34]. As physical hyperactivity frequently occurs in AN patients, who exhibit severe loss of both fat and lean mass, the potential of BCAA to prevent muscle waste or to enhance muscle gain appears to have a high relevance.

Thus, we aimed to evaluate the effects of glutamine and branched chain amino acids supplementation on food intake, body weight and composition and on intestinal functions in the rodent ABA model.

## 2. Material and Methods

### 2.1. Experimental Procedure

Protocol was approved by the regional ethical committee named CENOXEMA (authorization on N/05-11-12/28/11-15). To evaluate the potential benefits of Gln or BCAA in AN, we separately performed distinct experiments following ABA protocol with Gln or BCAA supplementation. For this, 8-week-old C57BL/6 male mice (Janvier Labs, Le Genest-Saint-Isle, France) were placed in individual cages and fed with a standard diet. Mice were acclimatized during 5 days at 23 °C with a reversed 12 h light–dark cycle (dark phase: 10:00 to 22:00) and then randomized in two groups: ad libitum (CTRL) and activity-based anorexia (ABA). The ABA experimental procedure was the same as described in our previous studies [14]. Briefly, ABA mice were placed in cages equipped with an activity wheel connected to Running Wheel^®^ software (Intellibio, Seichamps, France) while CTRL were placed in standard cages. After the acclimatization period, food access was progressively reduced for ABA mice from 6 h at day 6 to 3 h at day 9 and until the end of the experiment at day 17. Food was given when the dark phase started at 10:00. All mice had free access to water. In accordance with the ethical procedure, if the weight loss exceeded 20% within 3 consecutive days, animals were euthanized. Body weight, physical activity and food intake were monitored each day before the beginning of the dark phase.

At day 11, ABA mice were randomized and divided into two sub-groups with or without supplementation. Gln was distributed in water at the dose of 2 g/kg/day, as previously described [35]. Like Gln, BCAA were diluted in water, but consisted of a combination of three amino acids with a ratio of 2:1:1 (Leu, Ile, Val) to reach 25 g/L (2.5%) [36]. At last, concentrations for each BCAA were: 1.25% (Leu), 0.625% (Ile) and 0.625% (Val). Both Gln and BCAA beverages were renewed each day. Fluid intake was monitored each day by weighting drinking bottles. At day 17, mice received an intraperitoneal injection of 100 µL PBS containing puromycin (Sigma Aldrich, Saint Quentin Fallavier, France) at a dose of 0.040 µmol/g 20 min before euthanasia with a ketamine/xylazine solution, as previously described [21]. Blood was collected and stored in heparin-coated collecting tubes. All collected samples (jejunum, colon, hypothalamus), except blood, were immediately dropped in liquid nitrogen and the time spent since puromycin injection was recorded for protein synthesis rate calculation. To collect plasma, blood samples were centrifuged at 3000 *g* at 4 °C for 20 min. Then, plasma was removed and stored at −80 °C.

### 2.2. Body Composition

Whole body composition was assessed on vigil animals at day 17 using Minispec LF110 (Brucker, Wissembourg, France), a fast nuclear magnetic resonance method.

### 2.3. Evaluation of Intestinal Permeability

Colonic paracellular permeability was assessed by measuring fluxes of FITC-Dextran (4 kDa) in Ussing chambers (Harvard Apparatus, Holliston, MA, USA), as previously described [35]. Briefly, a physiological solution (HBSS) was placed in mucosal and serosal sides. After equilibration, FITC-Dextran (5 mg/mL) was added in the mucosal side. After 3 h at 37 °C, solution in the serosal side was collected and stored at −80 °C. Then, 100 µL of each sample was dropped in a black 96-well plate and the fluorescence level of FITC-dextran was measured with a spectrometer (excitation: 485 nm; emission: 535 nm) Chameleon V (Hidex, Turku, Finland). Values were converted into concentration (mg/mL) using a standard curve.

### 2.4. Protein Extraction and Western Blotting

Tissues were homogenized in 400 µL of ice-cold lysis buffer (200 µL A2X buffer, 2 μL dithiothreitol 100 mM, 50 μL NP40 1%, 1 μL protease inhibitors P8340, 2 μL phosphatase inhibitors P2850, for H_2_O 400 μL) using TissueLyser LT (Qiagen, Courtabœuf, France). Then, samples were placed on ice for 20 min and centrifuged for 15 min at 12,000 *g* at 4 °C. Supernatants were collected and protein concentrations were assessed using the Bradford method (excitation: 485 nm; emission: 595 nm) [37]. Total protein samples (25 µg) were separated by electrophoresis on 4–20 % gradient polyacrylamide gels (Biorad, Marnes-la-Coquette, France) for 30 min at 200 V and then transferred to a nitrocellulose membrane. The membranes were blocked for 2 h at room temperature with 5% BSA in Tris-buffered saline (10 mmol/L Tris, pH 8; 150 mmol/L NaCl) plus 0.2% Tween 20 (TBST). Then, membranes were incubated overnight at 4 °C with primary antibody and washed three times for 5 min in TBST followed by incubation with appropriate horseradish peroxydase (HRP) conjugated secondary antibody (1:5000 dilution, Dako, Santa Clara, CA, USA) for 1 h at room temperature. Afterwards, blots were washed three times for 5 min in TBST and immunocomplexes were revealed by using the enhanced chemiluminescence (ECL) detection system (GE Healthcare, Orsay, France). Protein bands were scanned (ImageScanner III; GE Healthcare) and quantified using Image-Quant TL software (GE Healthcare). Protein levels were normalized with ß-actin (1:5000 dilution, A5441, mouse monoclonal antibody, Merck, Darmstadt, Germany).

### 2.5. Protein Synthesis Analysis by the SUnSET Method

Surface sensing of translation (SUnSET) is a non-radioactive method used for in vivo assessment of total protein synthesis [38]. Puromycin was integrated in peptide chain and formed puromycin-conjugated peptides, which were revealed by Western blotting as described above by using a specific mouse monoclonal anti-puromycin antibody (MABE343, clone 12D10, 1:5000; Merck Millipore, Darmstadt, Germany). To determine the total protein synthesis rate, the densitometry of each whole lane was assessed. Results were expressed as the ratio puromycin expression/min, as previously described.

### 2.6. RT-qPCR

Extraction of colonic and hypothalamic total RNAs, quantification and reverse transcription were performed, as previously described [39]. Then, qPCR was completed by SYBR Green technology on Bio-Rad CFX96 real-time PCR system (Bio-Rad Laboratories, Marnes la Coquette, France) for the following genes: *MUC-2*, *CLDN-1*, *CLDN-2*, *ZO-1*, *OCLDN*, *NPY*, *POMC*, *IL-10*, *TNF-α*, *IL-1β*, *GAPDH*, *β2M*, *18S* (Table S1). The mean of GAPDH, β2M and 18S, used as housekeeping genes, was calculated and used as reference. To convert cycle threshold values into concentration values, we used the mRNA absolute quantification based on standard curves.

### 2.7. Adiponectin and Leptin Plasma Concentrations

Adiponectin and leptin plasma levels were assessed by using Milliplex 96-well plate multiplex assay MADCYMAG-72K (Merck Millipore, Darmstadt, Germany) following supplier instructions and using a laser-based detection instrument, the Bioplex 2200 (BioRad Laboratories, Hercules, CA, USA).

### 2.8. Colonic Intracellular Signaling Pathways

RayBio^®^ C-series Human and Mouse MAPK Pathway Phosphorylation Array C1 kits (RayBiotech, Tebu-bio, Le Perray en Yvelines, France) were used to measure the level of 17 proteins implicated in intracellular signaling. Briefly, nitrocellulose membranes were incubated for 2 h at room temperature in blocking buffer. Total colonic proteins (250 µg) were diluted in 5 mL of blocking buffer (50 µg/mL). Membranes were incubated for 4 h in diluted samples at room temperature. After washes, membranes were incubated overnight at 4 °C with detection antibody cocktail. After additional washes, HRP-Anti-Rabbit IgG (1:1000) was added for 2 h at room temperature. Chemiluminescence was detected on Amersham Hyperfilm ECL (GE Healthcare, Orsay, France). Spots were scanned (ImageScanner III; GE Healthcare) and quantified using Image-Quant TL software (GE Healthcare). Protein levels were normalized with the mean of positive control spots.

### 2.9. Statistical Analysis

Data were analyzed with GraphPad Prism 5.0 (GraphPad Software Inc., San Diego, CA, USA) and expressed as mean ± standard error mean (SEM). Comparisons between two groups were performed using the unpaired two-tailed Student’s *t*-test or non-parametric Mann–Whitney test, as appropriate. Concerning comparisons between three groups, differences were assessed using one-way ANOVA followed by Dunnet’s multiple comparisons as a post hoc test; or two-way ANOVA followed by Bonferroni as a post hoc test. A value of *p* < 0.05 was considered as significant.

## 3. Results

### 3.1. Body Weight and Body Composition

**Figure 1:**
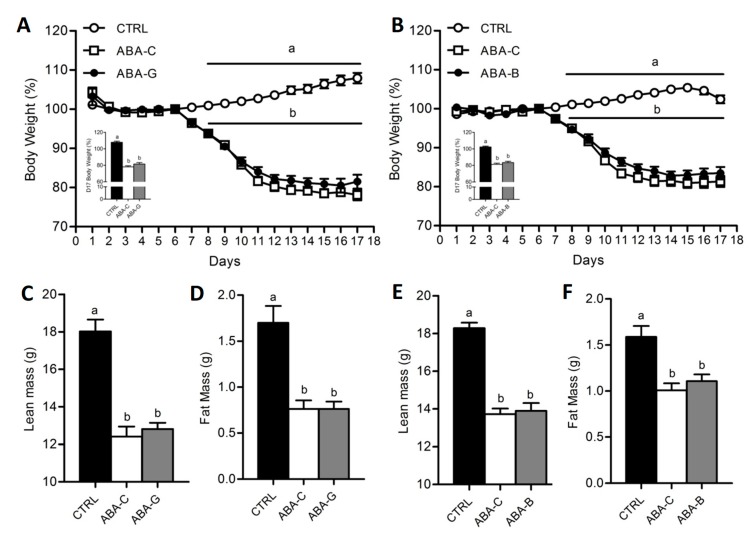
Body weight, lean mass and fat mass. Body weight was monitored each day, expressed in percentage compared to day 6, for both Gln (**A**) and BCAA (**B**) series with control mice (CTRL; open circles), non-supplemented activity-based anorexia mice (ABA-C; open squares, *n =* 16) and supplemented activity-based anorexia mice (ABA-G or ABA-B; closed squares). Body change at day 17 was measured, expressed in percentage compared to day 6, for both Gln and BCAA. Lean and fat mass, expressed in grams, were measured by using Minispec for both Gln (**C**,**D**) and BCAA (**E**,**F**) series. Column bar graphs showed control mice (CTRL; black bars), non-supplemented activity-based anorexia mice (ABA-C; open bars) and supplemented activity-based anorexia mice (ABA-G or ABA-B; grey bars). Values without a common letter significantly differ (*p* < 0.05). The number of mice was *n =* 16 for CTRL, *n =* 16 for ABA-C and *n =* 12 for ABA-G (**A**,**C**,**D**) and *n =* 16 for CTRL, *n =* 16 for ABA-C and *n =* 14 for ABA-B (**B**,**E**,**F**).

As observed in our previous studies [14,20], there was no significant difference between all groups from day 1 to day 6 (Figure 1A,B). However, once the food access was reduced, body weight significantly decreased in all ABA groups from day 8. At day 17, body weight loss was about 19–22% in ABA-C groups compared to day 6 (*p* < 0.05). Gln and BCAA supplementation did not affect body weight. ABA groups exhibited altered body composition with a decrease of both lean and fat mass (Figure 1C–F) without Gln and BCAA effects. ABA-C mice exhibited altered physical activity pattern. However, neither Gln nor BCAA supplementation affected physical activity.

### 3.2. Food Intake and Hypothalamic Neuropeptides

**Figure 2:**
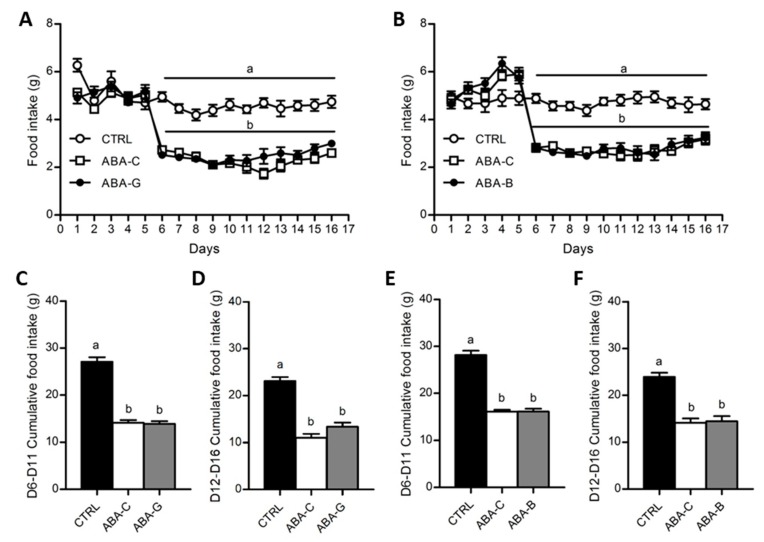
Food intake and cumulative food intake. Food intake was monitored each day, expressed in grams, for both Gln (**A**) and BCAA (**B**) series with control mice (CTRL; open circles), non-supplemented activity-based anorexia mice (ABA-C; open squares) and supplemented activity-based anorexia mice (ABA-G or ABA-B; closed squares). Cumulative food intake, expressed in grams, was calculated from day 6 to day 11 and from day 12 to day 16 (supplementation period) for both Gln (**C**,**D**) and BCAA (**E**,**F**) series. Column bar graphs showed control mice (CTRL; black bars), non-supplemented activity-based anorexia mice (ABA-C; open bars) and supplemented activity-based anorexia mice (ABA-G or ABA-B; grey bars). Values without a common letter significantly differ (*p* < 0.05). The number of mice was *n =* 16 for CTRL, *n =* 16 for ABA-C and *n =* 12 for ABA-G (**A**,**C**,**D**) and *n =* 16 for CTRL, *n =* 16 for ABA-C and *n =* 14 for ABA-B (**B**,**E**,**F**).

Food intake was daily monitored for both Gln and BCAA series (Figure 2A,B). From day 1 to day 6, food consumption was similar in all groups. Since day 6, food intake was progressively decreased in ABA groups until day 9 when the food intake of mice was reduced by nearly 2 times compared to the control groups (*p* < 0.05). Then, neither Gln nor BCAA supplementation significantly affected daily food intake until day 16. Cumulative food intake during food limitation until the beginning of supplementation (from day 6 to day 11) was reduced in ABA groups (Figure 2C,E) in the same way as described above. However, during the supplementation period (from day 12 to day 16), we observed a trend for an increase of cumulative food intake in the ABA-G group compared with the ABA-C group (*p* < 0.1) that was not observed for the ABA-B group (Figure 2D,F). We thus analyzed the hypothalamic mRNA levels of orexigenic neuropeptide Y (NPY) and anorexigenic pro-opiomelanocortin (POMC) neuropeptides. The NPY mRNA level, in both series, was similarly increased in supplemented and non-supplemented ABA groups compared with controls (*p* < 0.05). The mRNA level for POMC was only significantly decreased in ABA groups in the BCAA experiment. However, neither Gln nor BCAA affected POMC mRNA expression.

### 3.3. Colonic Permeability and Tight Junction mRNA

**Figure 3:**
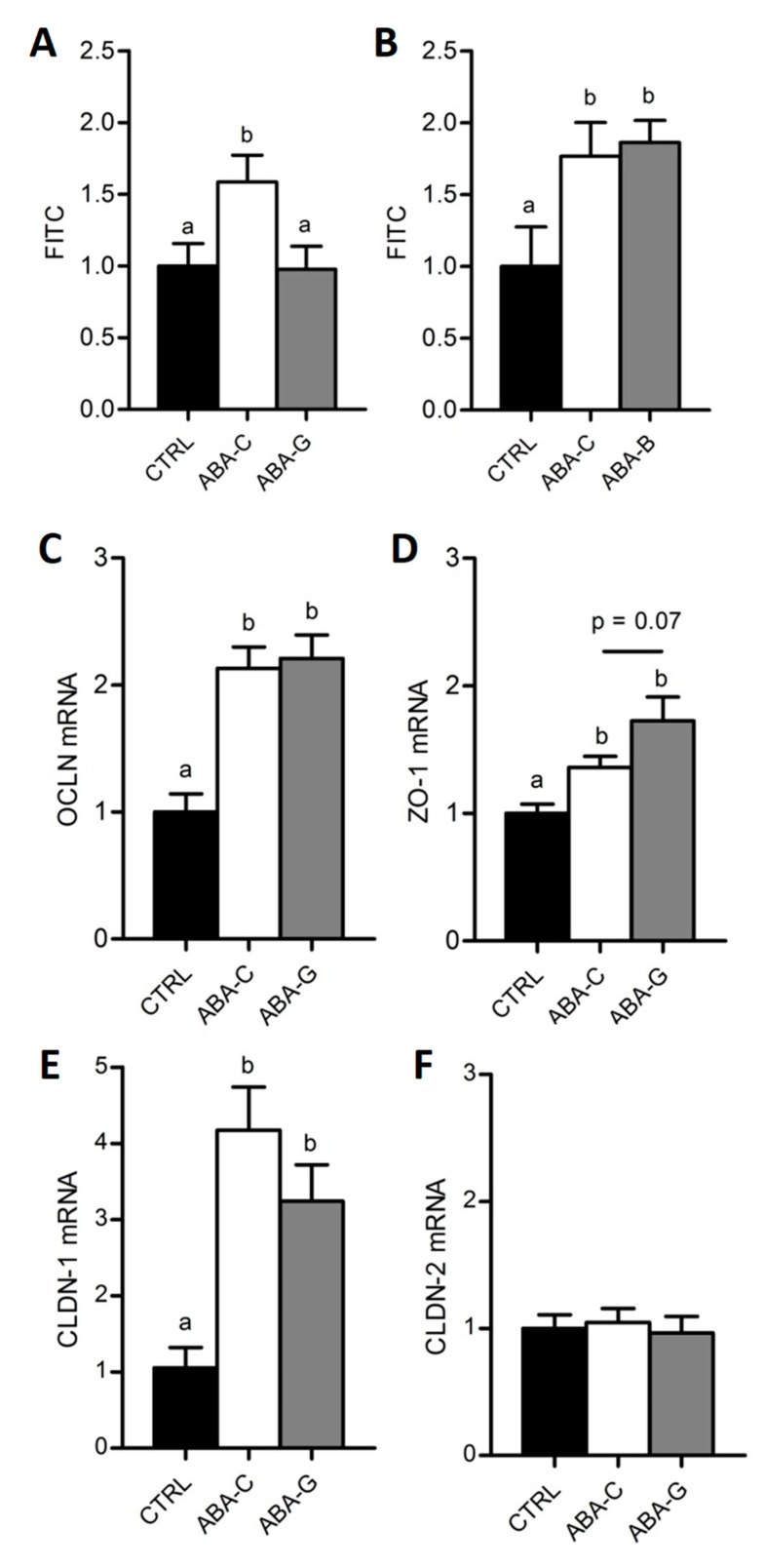
Colonic permeability and tight junction protein mRNA. Colonic paracellular permeability was evaluated in Ussing chambers with FITC-Dextran (4 kDa) in both Gln (**A**) and BCAA (**B**) series. Colonic tight junction protein mRNA levels were only assessed for Gln by using RT-qPCR: OCLN (**C**), ZO-1 (**D**), CLDN-1 (**E**) and CLDN-2 (**F**). Column bar graphs showed control mice (CTRL; black bars), non-supplemented activity-based anorexia mice (ABA-C; open bars) and supplemented activity-based anorexia mice (ABA-G or ABA-B; grey bars). Values without a common letter significantly differ (*p* < 0.05). The number of mice was *n =* 15 for CTRL, *n =* 15 for ABA-C and *n =* 12 for ABA-G (**A**), *n =* 8 for CTRL, *n =* 8 for ABA-C and *n =* 7 for ABA-B (**B**) and *n =* 16 for CTRL, *n =* 16 for ABA-C and *n =* 12 for ABA-G (**C**–**F**).

Here, we used FITC-Dextran to assess the paracellular permeability in colonic segment. In both Gln (Figure 3A) and BCAA (Figure 3B) experiments, colonic permeability was higher in ABA-C groups compared with control groups (*p* < 0.05). Interestingly, the ABA-G group exhibited a restoration of colonic permeability to control level (Figure 3A) that was not observed in the ABA-B group (Figure 3B), suggesting a specific beneficial effect of oral Gln supplementation on colonic permeability in the ABA model.

As Gln was able to limit colonic hyperpermeability in ABA mice, we assessed the level of mRNA coding for tight junction proteins in the Gln experiment. Concerning occludin (OCLN), claudin-1 (CLDN-1) and zonula occludens-1 (ZO-1), mRNA levels were increased in the ABA groups compared to the control group (*p* < 0.05) but there was no significant difference between Gln-supplemented and non-supplemented mice (Figure 3C–E). We observed a trend for an increase in the ZO-1 mrNA level in the ABA-G group compared with the ABA-C group, but the difference did not reach significance (*p* = 0.07). No difference occurred in any groups for claudin-2 (CLDN-2) mRNA (Figure 3F).

### 3.4. Mucin-2 mRNA and Colonic Protein Synthesis

**Figure 4:**
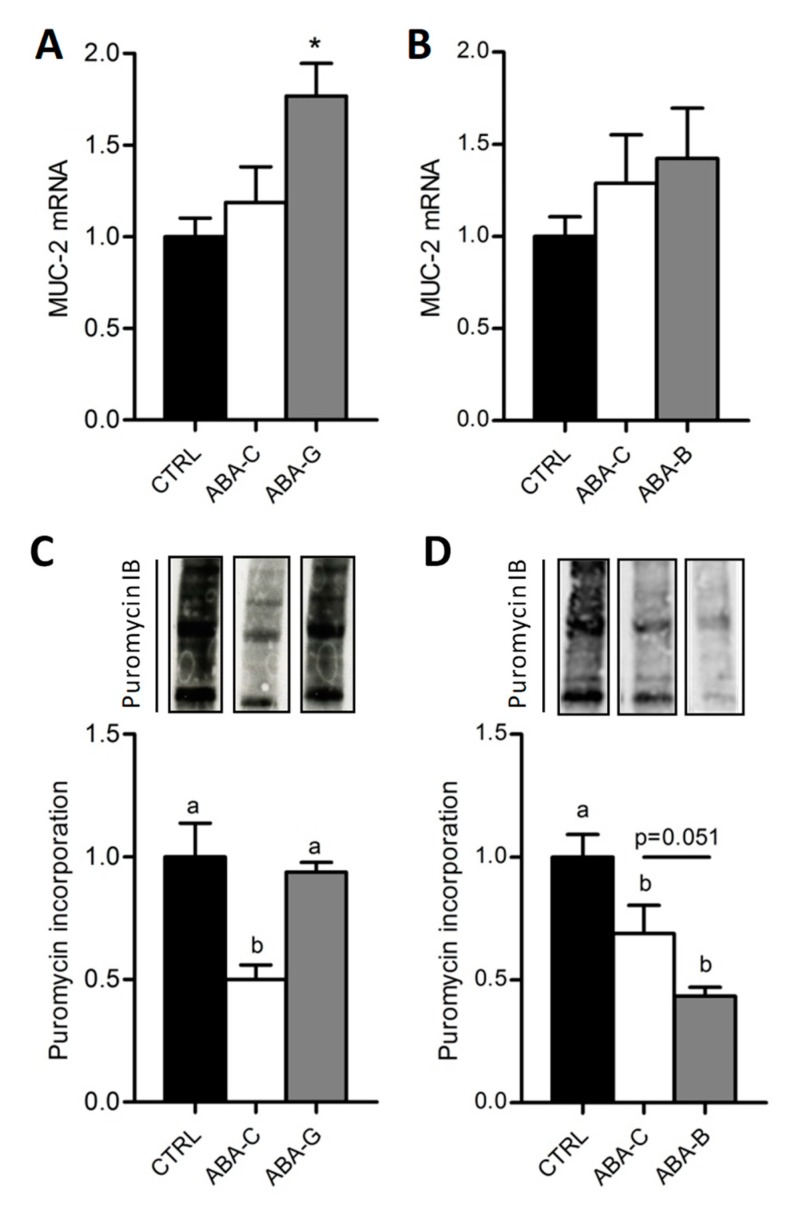
Colonic MUC-2 mRNA levels and total colonic protein synthesis. Colonic MUC-2 mRNA levels were evaluated by RT-qPCR in both Gln (**A**) and BCAA (**B**) series. The total colonic protein synthesis rate was measured with the SUnSET method in Gln (**C**) and BCAA (**D**) series with puromycin incorporation. Column bar graphs show control mice (CTRL; black bars), non-supplemented activity-based anorexia mice (ABA-C; open bars) and supplemented activity-based anorexia mice (ABA-G or ABA-B; grey bars). Values without a common letter significantly differ (*p* < 0.05); *: *p* < 0.05 vs. ABA-C using *t*-test. The number of mice was *n =* 8 for CTRL, *n =* 5 for ABA-C and *n =* 5 for ABA-G (**A**,**C**) and *n =* 16 for CTRL, *n =* 16 for ABA-C and *n =* 14 for ABA-B (**B**,**D**).

The mucus layer plays a key role in the regulation of gut barrier function. We thus analyzed the mucin-2 (MUC-2) mRNA level, a major secretory mucin released by goblet cells. Control and ABA-C groups exhibited a similar level for MUC-2 mRNA (Figure 4A,B). Gln-supplemented mice showed a higher mRNA level for MUC-2 than ABA-C and Control mice (*p* < 0.05) (Figure 4A), whereas we did not observe any modification in BCAA-supplemented mice (Figure 4B), suggesting that only Gln was able to increase MUC-2 release.

We previously reported that the colonic protein synthesis rate was reduced in ABA mice compared with controls [21] by using the SuNSET method. In the present study, total colonic protein synthesis (Figure 4C) was reduced in ABA-C mice compared to controls in Gln and BCAA experiments (*p* < 0.05) but was increased in Gln-supplemented mice (*p* < 0.05). Total protein synthesis was restored in the colon of ABA-G mice to control the level. BCAA did not reproduce the Gln effect. In addition, protein synthesis remains similar between all groups in the jejunum. These results suggest that only Gln supplementation was able to restore the protein synthesis rate in the colon of ABA mice.

### 3.5. Colonic Inflammation and Plasmatic Adipose Tissue Markers

**Figure 5:**
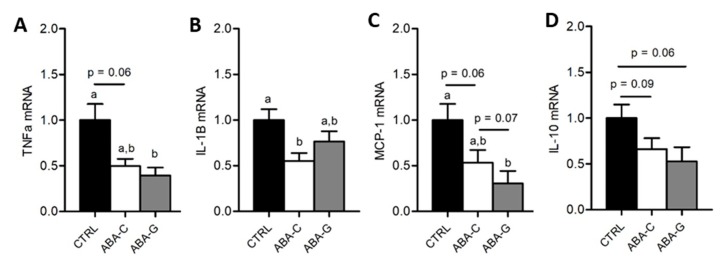
mRNA levels of colonic cytokines. The mRNA levels of colonic pro- and anti-inflammatory cytokines were evaluated by RT-qPCR for Gln experimentation: TNF-α (**A**), IL-1β (**B**), MCP-1 (**C**) and IL-10 (**D**). Column bar graphs showed control mice (CTRL; black bars, *n =* 16), non-supplemented activity-based anorexia mice (ABA-C; open bars, *n =* 16) and supplemented activity-based anorexia mice (ABA-G; grey bars, *n =* 12). Values without a common letter significantly differ (*p* < 0.05).

**Figure 6:**
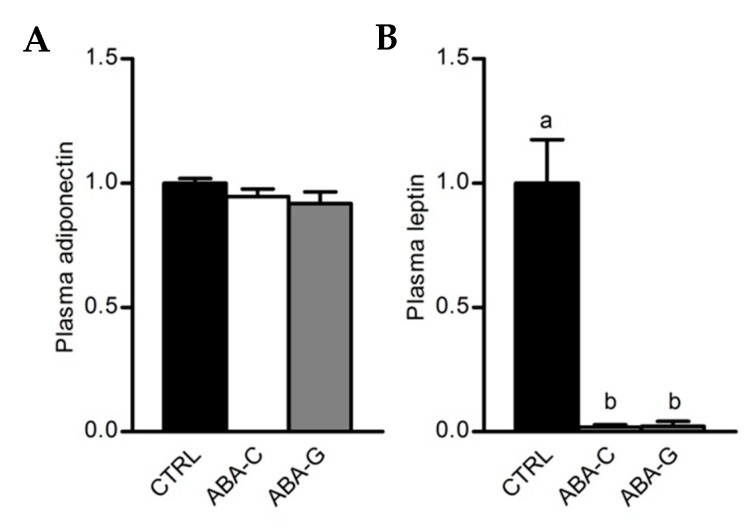
Plasmatic levels of adiponectin and leptin. Adiponectin (**A**) and leptin (**B**) plasmatic levels were measured for Gln supplementation test by multiplex assay. Column bar graphs show control mice (CTRL; black bars, *n =* 8), non-supplemented activity-based anorexia mice (ABA-C; open bars, *n =* 8) and supplemented activity-based anorexia mice (ABA-G; grey bars, *n =* 7). Values without a common letter significantly differ (*p* < 0.05).

As Gln was able to specifically restore protein synthesis and paracellular permeability in the colon of ABA mice and as Gln was previously reported to regulate intestinal inflammatory response [40,41], we assessed in the Gln experiment the mRNA levels for pro- and anti-inflammatory cytokines in colonic samples. The ABA-C group exhibited a decrease in TNFα and IL-1β mRNA levels (Figure 5A,B). Gln supplementation did not affect the TNFα mRNA level but partially restored IL-1β mRNA expression. For MCP-1, we observed a trend for a decrease in mRNA levels in the ABA-C group that reached significance in the ABA-G group (Figure 5C). For IL-10, we observed a trend for a decrease of mRNA levels both in ABA-C and ABA-G groups (Figure 5D). ABA groups also exhibited a marked decrease in plasma leptin with no modification of plasma adiponectin (Figure 6A,B). However, Gln and BCAA supplementation did not affect plasma adipokine concentration.

### 3.6. Signaling Pathways

**Figure 7:**
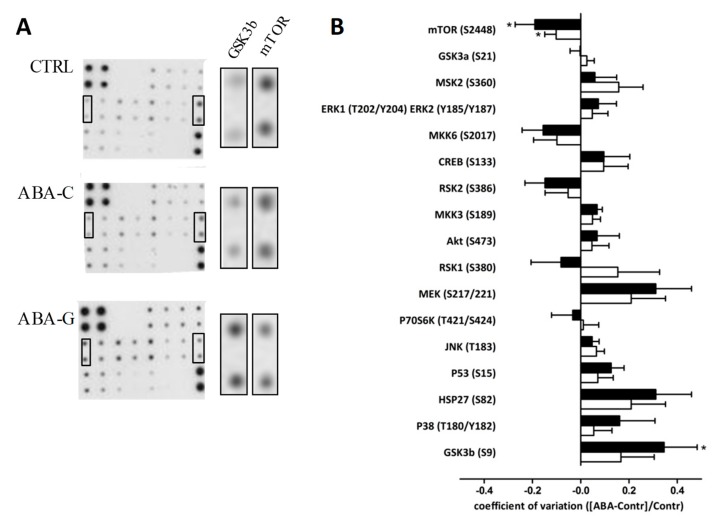
Colonic phosphorylation levels of proteins involved in signaling pathways. Phosphorylation levels of 17 proteins involved in the signaling pathway were evaluated by the MAPK array kit. Representative membranes are shown (**A**) for each group: CTRL, ABA-C and ABA-G, (*n =* 8 /group). Horizontal graph bar columns (**B**) show differential ratio levels in ABA-C (open bars) and ABA-G (black bars) compared to control mice. *: *p* < 0.05 vs. CTRL.

To better understand the mechanisms involved in the effects of Gln on protein synthesis and colonic permeability, we assessed the phosphorylation state of signaling proteins by using a macro-array kit. As shown in Figure 7, the level of phosphorylated mTOR was decreased in both ABA-C and ABA-G groups compared to control mice. However, phosphorylated GSK3b was increased in the ABA-G group but not in the ABA-C group. All other markers remained unaffected by Gln supplementation.

## 4. Discussion

Although AN is an eating disorder associated with severe body weight loss and frequent functional intestinal complaints that are only partially improved by refeeding, there is no specific treatment for AN. In addition, 9 to 52% of patients relapse after body weight restoration [42]. Over the last decade, the contribution of the gut–brain axis in the regulation of feeding and mood behaviors has emerged. We have thus evaluated the effects of specific amino acids: glutamine and brain chain amino acids in a mouse model of anorexia, the ABA model. Here, we demonstrate that oral Gln supplementation is able to specifically improve colonic parameters in ABA mice since BCAA supplementation remains inefficient. However, neither Gln nor BCAA affect body weight and composition.

In the present study, ABA mice exhibited severe body weight loss associated with a decrease in both lean and fat mass. Amino acids supplementation was not able to modify body weight and composition in our model. During other conditions such as ageing, BCAA and particularly leucine have been efficient in promoting muscle growth [34,43,44], even if other studies failed to demonstrate improved muscle mass after long-term BCAA supplementation [45]. Gln also failed to improve body weight and composition. In muscle, Gln represents nearly 50 to 60% of the total amino acid pool [46]. Under acute injury, muscle proteins are degraded, leading to Gln production. In anorectic patients, increased serum Gln has been reported, suggesting muscle protein degradation [47]. In our study, mice were not refed; this could explain the lack of BCAA and Gln effects by unmet energy needs. Regarding feeding behavior, we observed that only Gln was able to slightly increase cumulative food intake during the period of amino acids supplementation. ABA mice exhibited hypothalamic adaptive response with increased NPY and reduced POMC mRNA levels but neither Gln nor BCAA affected them. As the role of the gut–brain axis has been underlined in the control of feeding behavior, we focused on the colonic effects of amino acids.

As we previously reported [14,48], ABA mice showed an increase of approximately 50% in colonic permeability compared to control mice, which could be partially explained by undernutrition that is known to enhance intestinal hyperpermeability [49,50]. However, intestinal permeability in AN patients is poorly documented. Surprisingly, Monteleone et al. showed a reduction in intestinal permeability in moderately undernourished AN patients [12]. In the ABA model combining physical hyperactivity, severe body weight loss and low food intake, colonic permeability was enhanced while jejunal permeability remained unaffected [14]. Interestingly, in the present study, oral Gln supplementation was able to restore colonic paracellular permeability to the control level; this was not observed for BCAA supplementation. In addition, a trend for an increase in ZO-1 mRNA was observed in the Gln-supplemented group compared with the not supplemented group (*p* = 0.07). Our data are in accordance with previous studies showing an improvement of gut barrier function by Gln in other pathophysiological conditions [24]. For instance, oral Gln supplementation restored jejunal permeability during mucositis in rats [51] and partially limited colonic hyperpermeability in stressed mice [35]. Randomized clinical trials evaluating the effects of oral Gln supplementation also showed reduced intestinal permeability in patients with irritable bowel syndrome [29] or in chemotherapy-treated patients [52,53]. In Zhou’s study, whey proteins were used as a control underlying the specificity of Gln effects as in the present study. Then, to better understand the mechanisms by which Gln regulates colonic permeability, we evaluated other parameters such as MUC-2 mRNA or colonic protein synthesis.

MUC-2 is a major secretory mucin contributing to the mucus layer. In ABA supplemented with Gln, MUC-2 mRNA was increased compared with controls while BCAA did not reproduce these results. During parenteral nutrition, Gln enhanced the production of MUC-2 mRNA in the intestinal tract [28]. In burned rats, intragastric Gln infusion restored MUC-2 to control levels while MUC-2 was reduced in burned rats receiving intragastric alanine [54]. In the present study, BCAA did not affect MUC-2 whereas Mao et al. showed that leucine was able to improve MUC-2 synthesis in human colonic epithelial cell LS174T via the PI3K–Akt–mTOR pathway [55].

Finally, we evaluated total protein synthesis because ABA mice had a reduced protein synthesis in the colonic mucosa in a previous study [21] and both Gln and BCAA stimulated protein synthesis in intestinal tissues in different conditions [27]. In the present study, colonic protein synthesis was reduced in ABA mice, probably through a decrease in p70S6 kinase phosphorylation, as previously reported [21]. Oral Gln supplementation restored protein synthesis to control levels but did not affect p70S6 kinase. However, Gln-supplemented animals exhibited an increase in phosphorylated GSK3b, suggesting an inactivation of GSK3b that has been recently shown to favor glutaminolysis and thus glutamine utilization as a carbon source in lung cancer cells [56]. On the contrary, BCAA supplementation did not affect colonic protein synthesis in the present study, showing a specific effect of Gln. In Caco-2 cells, leucine supplementation increased protein synthesis through the mTOR pathway [57] that was not observed in the duodenal mucosa of healthy volunteers [58]. All these data underline that oral Gln improves colonic metabolism and colonic barrier function. It should be thus of interest to further evaluate putative intestinal disorders (motility, visceral sensitivity) that are frequent in AN patients [9,59] but that remains poorly documented in an animal model of AN. As observed in AN patients, gastric emptying was delayed in the ABA mice [60] but colonic functions should be further investigated.

Our study has some limitations. First, we used a middle-term ABA model, as previously described [14,20,21,48,60,61]. Initially, the ABA model was developed according to a short-term protocol [62,63]. More recently, a modified ABA protocol has been reported, leading to long-term starvation [64,65]. It should thus be interesting to evaluate nutritional interventions in those models that mimic metabolic disturbances observed in AN patients. Second, we did not include, in our study, food restricted and active control groups because we mainly aimed to evaluated the effects of amino acids in ABA mice but also because of ethical considerations. Finally, we were not able to describe the detailed mechanisms of action of glutamine on colonic barrier function. Recently, AMPK activation has been reported to enhance enterocytes polarization and ameliorate the assembly of tight junction proteins, leading to an improvement of intestinal barrier function [66], probably through the AMPK–CDX2 pathway [67]. The role of AMPK in the regulation of gut barrier function in ABA mice and its modulation by glutamine requires further investigation.

In conclusion, in a mouse model mimicking AN, oral glutamine supplementation ameliorates barrier function and protein synthesis in the colon without affecting body weight and composition. Those effects were not reproduced by branched-chain amino acids. Further studies should be conducted to evaluate the impact of Gln on intestinal disorders and to confirm these data in AN patients.

## Supplementary Materials

The following are available online at https://www.mdpi.com/2072-6643/11/6/1348/s1, Table S1: Primers sequences used for qPCR.
