# Supplementary material for: Glutamine, but not Branched-Chain Amino Acids, Restores Intestinal Barrier Function during Activity-Based Anorexia
Source: Nutrients. 2019 Jun 15;11(6):1348. doi: 10.3390/nu11061348 (PMC6628073; doi:10.3390/nu11061348)
Supplement: Supplementary file 1 [file nutrients-11-01348-s001.pdf]

Table S1. Primers sequences used for qPCR.

Genes	Sens	Sequences
18S	F	5'-TGCGAGTACTCAACACCAACA-3'
	R	5'-TTCCTCAACACCACATGAGC-3'
β2M	F	5'-GCCGAACATACTGAACTGCTAC-3'
	R	5'-GCTGAAGAACATATCTGACATCTC-3'
CLDN-1	F	5'-CTGGGTTTCATCCTGGCTTC-3'
	R	5'-TTGATGGGGGTCAAGGGGTC-3'
CLDN-2	F	5'-ATACTACCCTTTAGCCCTGACCGAGA-3'
	R	5'-CAGTAGGAGCACACATAACAGCTACCAC-3'
GAPDH	F	5'-CATCACTGCCACTCAGAAGA-3'
	R	5'-AAGTCACAGGAGACAACCT-3'
IL-1β	F	5'-CCCAAAAGATGAAGGGCTGC-3'
	R	5'-AAGGTCCACGGGAAAGACAC-3'
IL-6	F	5'-CACTTCACAAGTCGGAGGCT-3'
	R	5'-CTGCAAGTGCATCATCGTTGT-3'
IL-10	F	5'-ACCTGGTAGAAGTGATGCCC-3'
	R	5'-GCTCCACTGCCTTGCTCTTAT-3'
MCP-1	F	5'-TTAAAAACCTGGATCGGAACCAA-3'
	R	5'-GCATTAGCTTCAGATTTACGGGT-3'
MUC-2	F	5'-CGACACCAGGGATTTCGCTTAAT-3'
	R	5'-CACTTCCACCCTCCCGGCAAAC-3'
NPY	F	5'-CTGCGACACTACATCAATCT-3'
	R	5'-CTTCAAGCCTTGTTCTGG-3'
OCLN	F	5'-AGACTACACGACAGGTGGGG-3'
	R	5'-CTGCAGACCTGCATCAAAAT-3'
POMC	F	5'-CCTCCTGCTTCAGACCTCCA-3'
	R	5'-GGCTGTTCATCTCCGTTGC-3'
TNF-α	F	5'-TGTCTACTCCTCAGAGCCCC-3'
	R	5'-TGAGTCCTTGATGGTGGTGC-3'
ZO-1	F	5'-GCAGACTTCTGGAGGTTTCG-3'
	R	5'-CTTGCCAACTTTTCTCTGGC-3'
